# Drug Release Kinetics and Front Movement in Matrix Tablets Containing Diltiazem or Metoprolol/*λ*-Carrageenan Complexes

**DOI:** 10.1155/2014/671532

**Published:** 2014-06-19

**Authors:** Ruggero Bettini, Maria Cristina Bonferoni, Paolo Colombo, Laura Zanelotti, Carla Caramella

**Affiliations:** ^1^Department of Pharmacy, University of Parma, Parco delle Scienze 27/A, 43124 Parma, Italy; ^2^Department of Drug Sciences, University of Pavia, Viale Taramelli 12, 27100 Pavia, Italy; ^3^Eratech Srl, Via Gandine 4, 29121 Piacenza, Italy

## Abstract

In this work we investigated the moving boundaries and the associated drug release kinetics in matrix tablets prepared with two complexes between *λ*-carrageenan and two soluble model drugs, namely, diltiazem HCl and metoprolol tartrate aiming at clarifying the role played by drug/polymer interaction on the water uptake, swelling, drug dissolution, and drug release performance of the matrix. The two studied complexes released the drug with different mechanism indicating two different drug/polymer interaction strengths. The comparison between the drug release behaviour of the complexes and the relevant physical mixtures indicates that diltiazem gave rise to a less soluble and more stable complex with carrageenan than metoprolol. The less stable metoprolol complex afforded an erodible matrix, whereas the stronger interaction between diltiazem and carrageenan resulted in a poorly soluble, slowly dissolving matrix. It was concluded that the different stability of the studied complexes affords two distinct drug delivery systems: in the case of MTP, the dissociation of the complex, as a consequence of the interaction with water, affords a classical soluble matrix type delivery system; in the case of DTZ, the dissolving/diffusing species is the complex itself because of the very strong interaction between the drug and the polymer.

## 1. Introduction

The interest in exploiting chemical interaction in controlled drug delivery has greatly increased in last years. In this respect, polyelectrolytes have been extensively evaluated as carriers for the controlled delivery of ionic drugs. In particular, water-soluble polymers are capable of binding with oppositely charged drugs which can be released by an ion-exchange process in presence of electrolytes [1, 2]. In comparison to resonates, these systems present the advantage that, once the drug is released, the polymer dissolves, leaving no residues. This allows obtaining soluble systems able to prolong drug release without tail effects and therefore with more constant release rate.

Based on this principle, different matrix systems were designed and thoroughly studied [3–5]. Swelling/release characteristics of tertiary amine or carboxylic acid pendent groups are however pH dependent and can vary with the pH in the gastrointestinal tract. In the case of basic drugs, less pH dependent release kinetics were reported from polymers containing sulfonate groups in the polyelectrolyte chain. In this perspective, some specifically designed methacrylate derivatives were proposed [6, 7]. Similar behavior, however, can be found in natural polysaccharides, such as carrageenans. The ionic interaction between lambda carrageenan and basic drugs in oral controlled release matrix tablets was proposed and characterized [8–10]. It was observed that different drugs give complexes with quite different characteristics of solubility and drug release kinetics [11, 12]. The different solubility of the complex results in fact in differences in water uptake and gelation properties. A different ability to form a hydrated gel layer around the matrix tablets was visually observed during the release test. The more soluble complex between carrageenan and metoprolol tartrate (MTP) showed a thick gel layer that can explain the diffusive drug release profiles, while the less soluble complex with diltiazem (DTZ) was characterized by a fast water uptake due only to capillarity, but no further gelation was observed. Techniques such as that developed by Bettini et al. [13] for studying the gel layer behavior during drug release seemed particularly suitable to make this aspect clearer. This technique allows visualizing the interfaces between dissolution medium and gel layer (erosion front) and between gel layer and not yet hydrated core of the matrix (swelling front) in a cylindrical matrix clamped between two transparent disks. Using this experimental setting, these authors clarified the mechanism of drug transport in pH-sensitive swelling controlled release systems [14].

In the present work, we investigated the front movement and the associated drug release kinetics in matrix tablets prepared with two complexes between *λ*-carrageenan and two soluble model drugs, namely, diltiazem HCl and metoprolol tartrate, respectively. We aimed at clarifying the role played by drug/polymer interaction on the water uptake, swelling, drug dissolution, and drug release performance of the matrix.

Dissolution and swelling/erosion experiments were performed both with the USP Apparatus 2 method and with the transparent disks method [13], in distilled water and in simulated gastric and intestinal fluids.

## 2. Materials and Methods

Physical mixtures between *λ*-carrageenan, CRG (Viscarin GP209, FMC, Prodotti Gianni, Milan, Italy), and diltiazem HCl, DTZ (Profarmaco, Milan, Italy) (40 : 60 w/w), or metoprolol tartrate, MTP (Moehs, Barcelona, Spain) (33 : 67 w/w), were prepared by blending the powders in a Turbula mixer (Bachofen AG, Germany) for 15 minutes. The adopted drug polymer ratios corresponded to the stoichiometry of the polymer/drug complex as previously determined by means of dialysis equilibrium studies [9]. For all powders, particle size fraction between 45 and 75 *μ*m was employed.

Two complexes between the drugs and CRG polymer were prepared by kneading a physical mixture of the components with water according to the method previously described [12]. Briefly, a mixture of 40 g of CRG and 60 g of diltiazem HCl or in alternative 33 g of CRG and 67 g of metoprolol was kneaded in a china mortar with distilled water (about 100 mL per 100 grams of powders) until a homogeneous paste was obtained. Then, the slurry was centrifuged; then, the solid phase was washed twice with distilled water in order to eliminate the soluble salts generated as by-product of the complex formation reaction (Cl^−^ and the inorganic cations of the *λ*-carrageenan) and dried in a ventilated oven at 40°C for 8 hours. The dried complex was sieved in order to separate the 45–75 *μ*m particle size fraction that was used throughout the experimentation. The actual drug content of the physical mixtures and the relevant kneaded products was determined. About 20 mg of powder accurately weighed was suspended in 200 mL of HCl 0.1 N, sonicated for 5 minutes (Branson 2200 Ultrasonic Cleaner, CT, USA), and kept under magnetic stirring for 24 hours at 37°C. After filtration, drug concentration of the solution was determined with a validated spectrophotometrical method at 236 and 274 nm for DTZ and MTP, respectively (V530, Jasco, Tokyo, Japan).

Cylindrical matrix tablets of 150 ± 0.2 mg weight and 3 ± 0.2 mm thickness were prepared by direct compression of either physical mixtures or complexes with a single punch tableting machine (EKO, Korsh, Germany), equipped with flat punches of 7 mm diameter at a compression force between 20 and 30 kN.

For comparison purpose, matrix tablets containing hydroxypropyl methylcellulose, HPMC (Methocel K100M CR Premium EP, Colorcon, UK), instead of CRG were prepared from DTZ-HPMC or MTP-HPMC physical mixtures in the same above reported conditions.

Drug release experiments were carried out at 37°C using a USP 34 Apparatus 2 (DT6 R, Erweka, Heusenstamm, Germany) with paddle rotating at 50 rpm, in either 1 liter of distilled water, phosphate buffer at pH 6.8 (Eur.Ph 7th), or simulated gastric fluid without enzymes at pH 1.2. The amount of drug released was determined spectrophotometrically at 236 nm for DTZ and 274 nm for MTP.

A second set of drug release experiments was conducted in order to follow, contemporary to drug release, matrix swelling and dissolution and matrix front movement, using the experimental setting previously reported [13]. Briefly, the matrix bases were clamped between two transparent Plexiglas discs (diameter 30 mm; thickness 5 mm). The assembled system was introduced into the vessel of dissolution apparatus containing 1 liter of medium at 37°C. In order to avoid boundary layer effects, a paddle rotation speed of 200 rpm was selected [15]. The matrix was video recorded through the transparent Plexiglas and the pictures, taken at fixed time intervals, were analyzed using ImageJ 1.43 software (NIH, USA) in order to measure the matrix swelling and the position of the fronts.

## 3. Results and Discussion

### 3.1. Diltiazem Release

**Figure 1:**
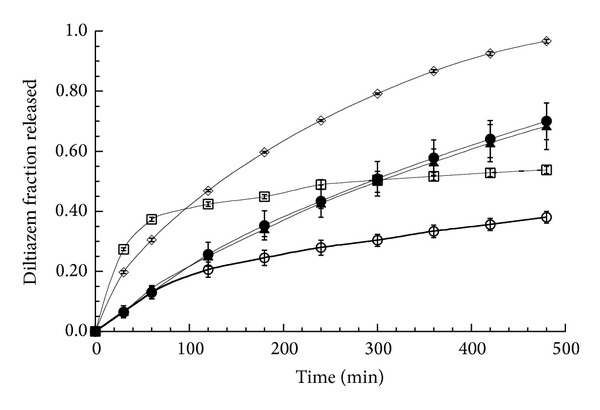
Fraction of diltiazem released as a function of time in different dissolution media from matrices consisting of DTZ-CRG complex in water (empty circle), DTZ-GRG physical mixture in water (square), DTZ-HPMC physical mixture in water (diamond), DTZ-CRG complex in pH 1.2 (solid circle), and DTZ-CRG complex in pH 6.8 (solid triangle). The bars represent the standard deviation (*n* = 3). Lines are simple interpolation of the experimental points.

In Figure 1, the diltiazem fraction released in distilled water versus time from DTZ-CRG matrices made with both the complex and the physical mixtures and from DTZ-HPMC matrices is reported. Drug release profiles of the matrices DTZ-CRG complex obtained in buffer pH 1.2 and pH 6.8 are also given.

By examining the profiles obtained in distilled water for matrices made with DTZ-CRG complex and DTZ-CRG physical mixture, a lower fraction of drug released after 8 hours compared to matrix containing DTZ-HPMC was observed. In particular, a slower and less variable release rate was observed when the matrix contained the complex (release rate between 0.2 and 0.05 mg/min) (release rate was calculated with the Derivative Macro of Kaleida Graph, Synergy Software (Reading. PA, USA). This macro finds the incremental slope of a curve, given the *x*-*y* data points describing the curve, and gives rise to a new curve. The slope is calculated using the two-point slope formula) while the matrix made with DTZ-CRG physical mixture presented a biphasic profile characterized by a rapid initial diltiazem release rate (nearly 0.8 mg/min) followed by a sudden decrease to a rate (0.02 mg/min) fairly similar to that of the matrix containing DTZ-CRG complex.

The profile relative to DTZ-CRG physical mixture matrix in the first 60 minutes was comparable as slope to that of the DTZ-HPMC matrix, whereas in the following time the release rate was similar to the DTZ-CRG complex matrix. Differently from CRG, HPMC is a nonionized polymer, unable to form ionic bonds with a weak base such as diltiazem. As a consequence, DTZ delivery control is mainly performed by diffusion through the polymeric HPMC swollen network.

The analysis of the drug release kinetics was performed by fitting the experimental data with the power equation [16]:

(1) $$\begin{array}{c} \frac{{\text{M}}_{t}}{{\text{M}}_{\infty }}=kt^{n}, \end{array}$$

where M_*t*_/M_*∞*_ is the fraction of drug released at time *t*, *k* is a kinetic constant, and *n* the diffusional exponent accounting for the type of kinetics observed. In the analysis proposed by Peppas [16], the *n*  value for a cylindrical geometry ranges between 0.45 and 0.89 according to the prevalence of pure Fickian or Case II transport, respectively, being the intermediate values indicative of an anomalous transport. The analysis performed on the data until 60% of drug released gave rise to *n* values of 0.61 (s.d. 0.07, *R* ^2^ 0.978) and 0.62 (s.d. 0.008, *R* ^2^ 0.999) for the matrices made with DTZ-CRG complex and DTZ-HPMC mixture, respectively; in both cases, anomalous diltiazem transport was deducted in agreement with previously published data [9, 17]. In the case of the matrix constituted by the physical mixture DTZ-CRG, the value obtained was strangely low (*n* = 0.22, s.d. 0.01, *R* ^2^ 0.965). This unusual value calculated is attributed to the particular shape of release profile that may indicate that the whole drug release was affected by some physicochemical phenomenon interfering with drug transport, thus impeding the correct application of the interpretative model. Similar behavior was reported by Tamimi et al. for the release of doxycycline from brushite cements [18]. Apart from the different polymers used for matrix formation, the dual release behavior of the DTZ-CRG physical mixture matrix must be attributed to different state of drug in the matrix [19]. In detail, the initial faster release is attributable to the free drug dissolution on the matrix surface and diffusion through the hydrated polymeric network; as the water penetrates the matrix of DTZ-CRG physical mixture, the formation* in situ* of the insoluble DTZ-CRG complex modifies the apparent solubility and the diffusional properties of DTZ, making the release rate similar to that of the matrix containing the DTZ-CRG complex. In summary, the peculiar shape of the release profile of matrix made with DTZ-CRG physical mixture reflects the transformation of the free drug in complex form with the polymer, being the interaction mediated by the water uptaken by the matrix.

Bonferoni et al. [19] reported that solubility and dissolution rate for CRG complex with DTZ were influenced by ionic strength. Furthermore, Naim et al. illustrated the role played by ions on swelling and erosion of *κ*-carrageenan matrices [20].

These previous reports prompted us to investigate the role of the medium pH end ionic strength on drug release from the CRG matrices.

Drug release profiles in buffered solution at both pH 1.2 (ionic strength 0.11 M) and pH 6.8 (ionic strength 0.47 M) from the complex are reported in Figure 1. The two curves were practically superimposed and almost linear, with *n* values of 0.89 (s.d. 0.07, *R* ^2^ 0.992) and 0.84 (s.d. 0.05, *R* ^2^ 0.995) for the medium at pH 1.2 and 6.8, respectively. The lack of difference and the linearity of the profiles are in agreement with previously published drug released data although the hydrodynamics of the dissolution experiments was different [9, 12, 19]. Compared to that obtained in water, the release profile obtained in the buffered solutions presented significantly higher release rate and more linear kinetics. In agreement with Bonferoni et al. [19], these data can be interpreted as the effect of the ionic competition between the drug and the cations in solution for the interaction with the polymer; this competition determines a displacement of the active ingredient from the complex once the latter is in a solution containing ionic species, thus facilitating the drug release.

### 3.2. Diltiazem Release and Front Position

Dissolution experiments with the two DTZ-CRG matrices were carried out also with the special device for the observation and the measurements of the matrix front position [13].

**Figure 2:**
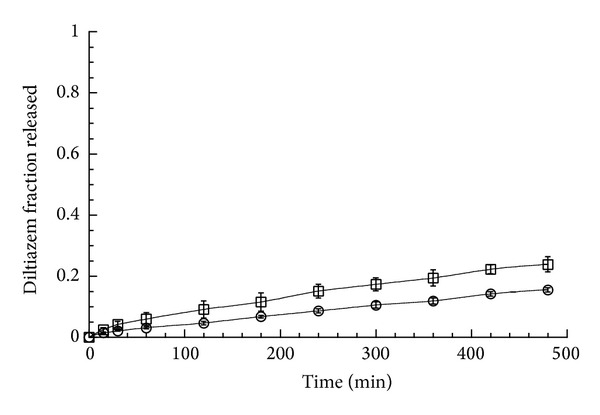
Fraction of diltiazem released as a function of time from matrices consisting of DTZ-CRG complex (circle) and DTZ-GRG physical mixture (square) and clamped in the special device for front position observation. The bars represent the standard deviation (*n* = 3).

Figure 2 reports the fraction of diltiazem released in water under these experimental conditions during the first 8 hours from the two CRG containing matrices. The relative difference between the release rate of the complex and the physical mixture mirrored that already observed in the USP Apparatus 2 even though the fractions released in this last case were nearly double. This is due to the smaller matrix surface available for drug release when the matrix bases are clamped between the two Plexiglas disks [13] as this experimental set-up allowed the water uptake and drug release only from the lateral side of the matrix. From the point of view of the mechanism, the release profile from the complex containing matrix was comparable to that observed in the USP Apparatus 2 experiments (*n* = 0.7, s.d. 0.007, *R* ^2^ 0.998).

On the other hand, the matrix containing the physical mixture did not show the biphasic profile observed when the drug dissolution was carried out without the Plexiglas device. The value of the obtained diffusional exponent *n* was 0.65 (s.d. 0.05, *R* ^2^ 0.997) suggesting a typical anomalous diffusion process. Obviously, the data presented in Figure 2 should be considered carefully as the amount of drug released in 8 hours was a small fraction of the total dose. However, when the experiments were prolonged for 24 hours, the same profile and kinetics were observed (data not shown). Overall, the matrix containing the physical mixture did not give rise to an initial burst release, likely because of the reduced releasing area exposed to the dissolution medium available for water uptake and for drug release. In this case, the release of the free drug from the matrix surface was minimized and the formation of the complex was more progressive.

**Figure 3:**
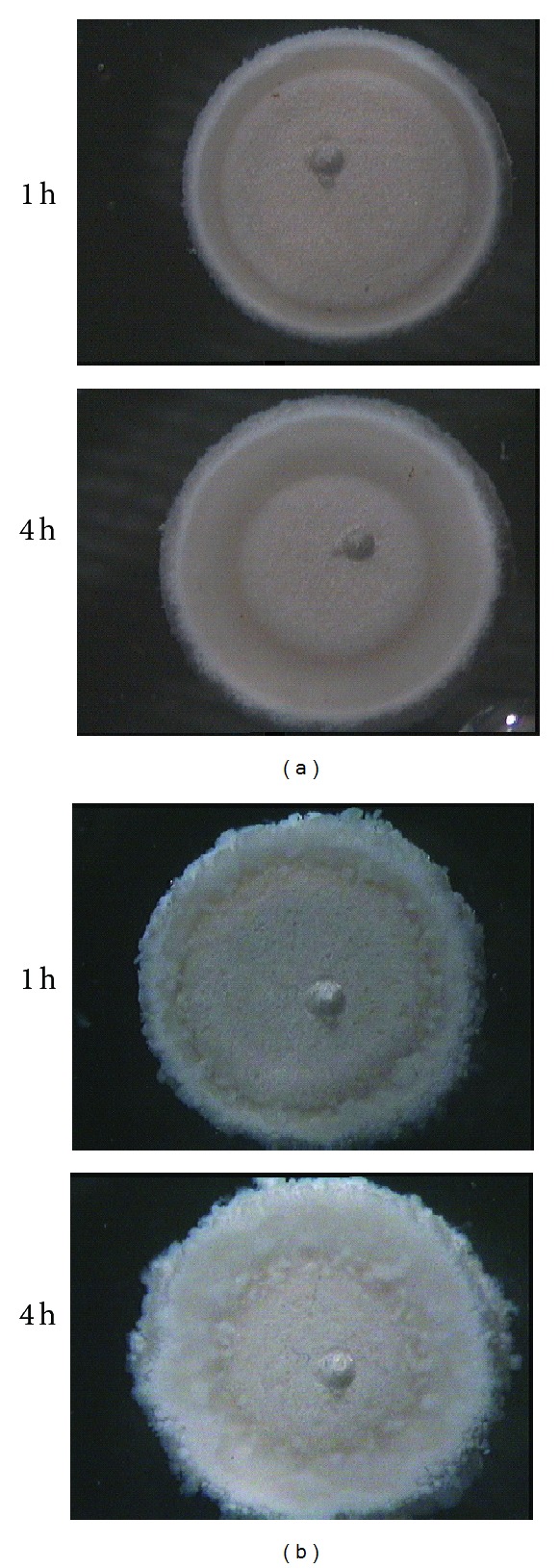
Images of the matrix clamped between two Plexiglas discs: DTZ-CRG complex (a) and DTZ-GRG physical mixture (b) taken after 1 and 4 hours of contact with water.

In Figure 3, as an example, pictures of the bases of both matrices taken through the transparent Plexiglas at 1 and 4 hours during the dissolution experiments carried out in water are shown.

As far as the matrix containing the complex is concerned (Figure 3(a)), three fronts are clearly observed since the early dissolution time. The front of the water (swelling front) penetrated progressively toward the matrix center, while the matrix front (eroding front) apparently maintained the same position. Interestingly, the presence of a third front (drug/complex diffusion front) that remained quite close to the eroding front can be observed.

It is worth underlining that up to now the diffusion front has been detected only in the presence of a colored diffusing species such as either the loaded drug itself [15] or when a die or an indicator was added in the dissolution medium [4, 21], while in the present case both the drug and the complex were white.

As to the CRG physical mixture containing matrix (Figure 3(b)), it can be observed that the matrix showed a remarkably different morphology with respect to that containing the complex. The progressive penetration of the solvent gave rise to a nonuniform gel layer with uneven outlines. This in practice impeded the clear identification of the front position. The observation of the progressive formation of an irregular gel layer, looking like an agglomeration of discrete particles, supported the hypothesis of the formation* in situ* of the nonsoluble complex.

**Figure 4:**
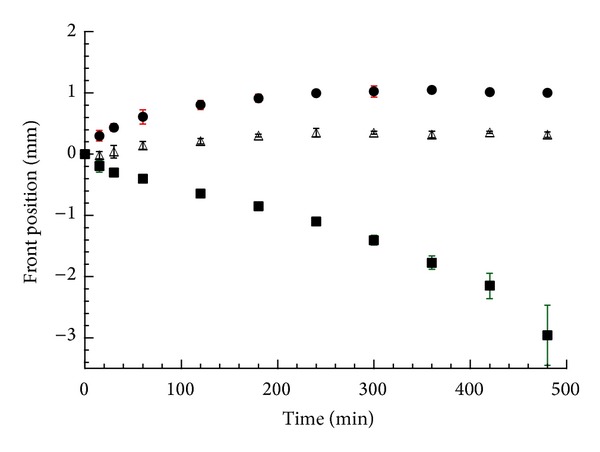
Position of the fronts as a function of time in the DTZ-CRG complex matrix in water: erosion front (circle), diffusion front (triangle), and swelling front (square). The bars represent the standard deviation (*n* = 3).

Figure 4 reports the front position versus time relevant to the matrices prepared from the complex. At the beginning of the experiments, the positions of the three fronts coincide and correspond to the point (0; 0) in the axis coordinates. The swelling front moved toward the matrix center almost linearly, while the position of the erosion front after an initial (2 hours) outwards movement leveled off. The diffusion front followed the movement of the erosion one making the dissolved drug gel layer thickness [15] almost constant after two hours. The front movement profile closely resembled that of a matrix containing a poorly soluble drug where the outward movement of the diffusion front was interpreted as the consequence of poorly soluble drug particle translocation due to polymer swelling [22].

As a matter of fact, the distance between the diffusion and the swelling front is inversely related to the drug solubility [22]. Therefore, the observed large distance between the diffusion and the swelling front testifies that the DTZ-CRG complex, whose solubility [11] is much lower than that of pure DTZ [23, 24], represents the diffusing species in the hydrated matrix.

The front behavior of the DTZ-CRG complex in buffered solutions at pH 1.2 or 6.8 was not substantially different (data not shown), with the only exception of the distance between the erosion and the diffusion front, namely, the dissolved drug gel layer thickness, which represents the diffusion path length that the drug molecules have to cover to reach the bulk solution.

**Figure 5:**
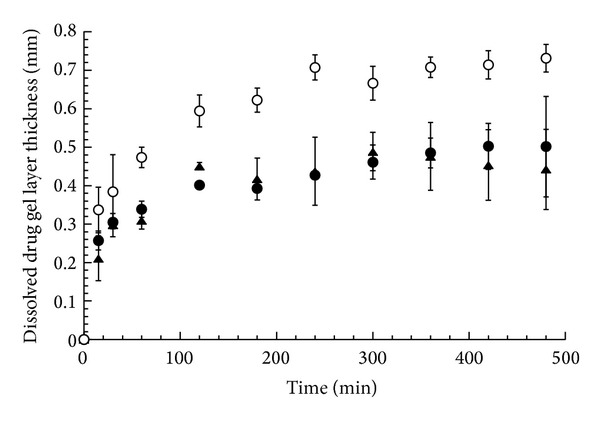
Dissolved drug gel layer thickness as a function of time in the DTZ-CRG complex matrix in water (circle), in pH 1.2 (solid circle), and in pH 6.8 (solid triangle). The bars represent the standard deviation (*n* = 3).

In Figure 5, the dissolved drug gel layer thickness measured in matrices of DTZ-CRG complex in water, pH 1.2, and 6.8 is plotted versus time. The dissolved drug gel layer obtained in water was thicker than that obtained in the buffered solution with the greatest increase in the first four hours followed by a very slow increment. The curves obtained in the buffered media overlapped each other with a profile leveling off after two hours. These constant and thinner gel layers justify the faster and more linear drug release profiles obtained in the buffered solution with respect to that in water (Figure 1) and can be explained by considering the different DTZ-CRG complex solubility in distilled water (0.86 mg/mL) with respect to the buffered media (2.2 and 1.79 mg/mL) in pH 1.2 and pH 6.8, respectively [11]. Colombo et al. [15] previously showed in HPMC matrices an inverse relationship between drug release rate and undissolved drug gel layer thickness stemming from the drug solubility difference.

### 3.3. Metoprolol Release

**Figure 6:**
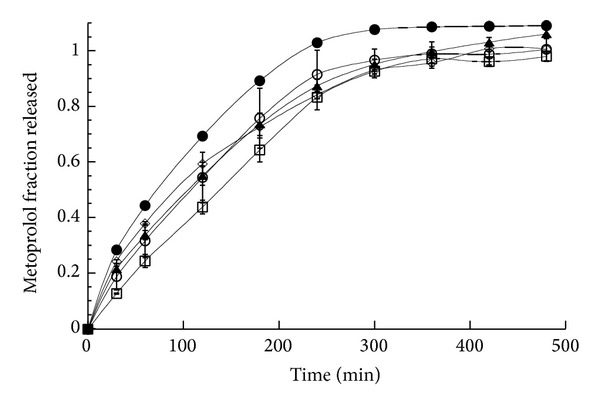
Fraction of metoprolol released as a function of time in different dissolution media from matrices consisting of MTP-CRG complex in water (empty circle), MTP-GRG physical mixture in water (square), MTP-HPMC physical mixture in water (diamond), MTP-CRG complex in pH 1.2 (solid circle), and MTP-CRG complex in pH 6.8 (solid triangle). The bars represent the standard deviation (*n* = 3). Lines are simple interpolation of the experimental points.

Figure 6 reports the MTP fraction released as a function of time in water for the complex, the MTP-CRG, and MTP-HPMC physical mixtures as well as for the complex in the two buffered media. In all cases, at least 80% of MTP dose was released in the first four hours. A slightly higher rate was recorded in pH 1.2, while the MTP-CRG physical mixtures in water showed the lowest rate, although not significantly different from the complex (both in water and in pH 6.8 buffer) and the MTP-HPMC physical mixture. Similar differences in MTP release profiles at pH 1.2 and 6.8 have been already reported by Aguzzi et al. [11] and can be probably ascribed to the possible higher solubility of the drug at highly acidic pH, stemming from the fact that MTP is a base in nature (pKa 9.7).

Table 1 summarizes the values of the exponent *n* and the relevant correlation coefficients obtained by fitting the drug release data reported in Figure 6 with (1).

Linear or quasilinear kinetics was obtained in water for the CRG containing matrices, while, in the buffered media, as well as for the HPMC matrix, anomalous kinetics was observed.

It is worth noting that this behavior is exactly the opposite of that obtained in the case of DTZ containing matrices. This suggests a differing sensitivity to the ion exchange process which is ascribed to the lower stability of the MTP-CRG relative to the DTZ-CRG complex.

In fact, the similarity between the release profiles of the MTP-CRG complex and the physical mixture, as opposite to DTZ, indicates that the interaction between MTP and CRG is overcome by that of MTP and water, indicating a weaker constant of formation/stability of the MTP-CRG complex relative to that of DTZ-CRG complex in water.

### 3.4. Metoprolol Release and Front Position

**Figure 7:**
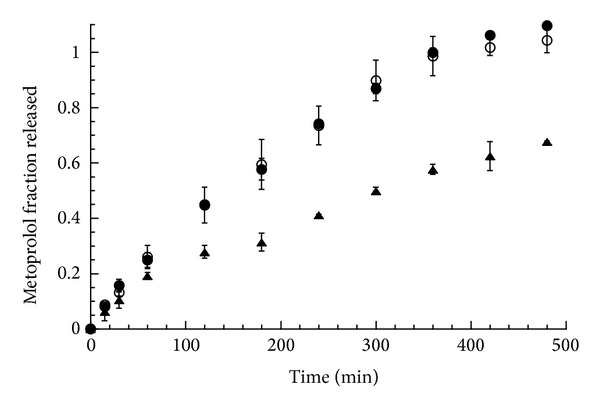
Fraction of metoprolol released as a function of time from MTP-CRG complex matrices clamped in the special device for front position observation, in water (empty circle), in pH 1.2 (solid circle), and in pH 6.8 (solid triangle). The bars represent the standard deviation (*n* = 3).

When the dissolution experiments were carried out on the MTP-CRG complex matrices with the Plexiglas device in water or in the buffered media (Figure 7), a complete drug release was obtained in 8 hours at pH 1.2 (*n* = 0.79, s.d. 0.0007, *R* ^2^ 0.996) and in water (*n* = 0.82, s.d. 0.09, *R* ^2^ 0.997), while at pH 6.8 a slower and less linear release (60% in 8 h) was observed (*n* = 0.69, s.d. 0.11, *R* ^2^ 0.988).

The swelling/dissolution behavior of these matrices revealed substantial difference with respect to the DTZ containing matrices. As in this last case, in the MTP matrices, the three fronts could be clearly distinguished; however, with the exception of pH 6.8 buffer, a progressive matrix dissolution was observed in the tested media.

**Figure 8:**
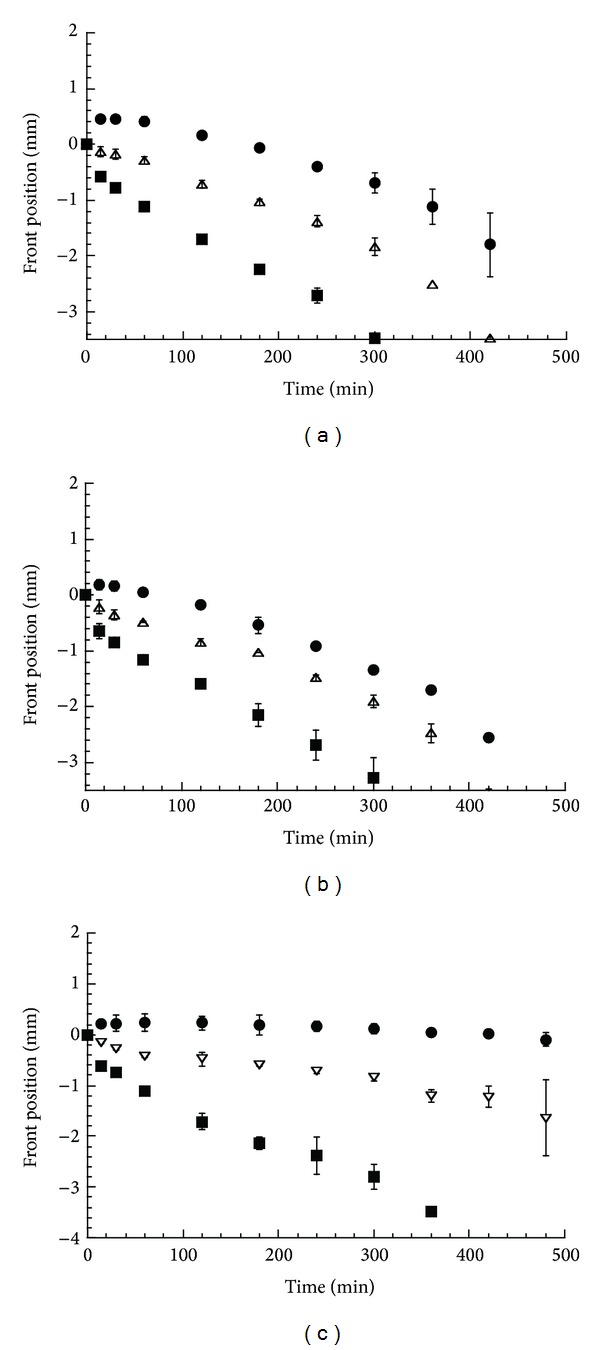
Position of the fronts as a function of time in the MTP-CRG complex matrix in water (a), pH 1.2 (b), and pH 6.8 (c): erosion front (circle), diffusion front (triangle), and swelling front (square). The bars represent the standard deviation (*n* = 3).

The measurement of the front position versus time provided more insights on the drug release behavior of these systems. In water (Figure 8(a)), the swelling front was fast and reached the matrix center in 5 hours. As already observed in matrices containing a soluble drug [15, 22], the diffusion front followed the swelling front but with lower rate, getting to the center of the matrix in about 7 hours. After an initial (half an hour) outward movement, the erosion front turned to move inward synchronizing its movement with that of the other two fronts. Similar patterns were observed for the experiment carried out in acidic pH (Figure 8(b)). In this case, however, the erosion front was closer to the diffusion front, affording a thinner dissolved drug gel layer and almost complete matrix dissolution in 8 hours.

The front synchronization and the consequent constant gel layer thickness explain the relatively high values (0.8) of the *n* exponent observed [25]. On the other hand, the matrix dissolution justifies the complete drug release in 8 hours.

In the phosphate buffer at pH 6.8 (Figure 8(c)), the erosion front maintained a fairly constant position, while the diffusion front moved inward though with remarkably lower rate than the swelling front. Therefore, no front synchronization occurred and the gel layer thickness progressively increased with time, thus accounting for the lower drug release rate and value of exponent *n* observed (Figure 7).

The different behavior in the phosphate buffer can be ascribed to the effect of the pH and the ionic strength on the matrix dissolution. The pH value of the dissolution medium affects in an opposite way the solubility of the drug and the polymer; for example, at pH 1.2, the fraction of dissociated drug is nearly 6 orders of magnitude higher than that at pH 6.8, while the polymer, being an acid in nature, is more soluble at the highest pH value.

Finally, the great difference observed between pH 1.2 and 6.8 buffer when the drug release was carried out with or without disk restriction can be justified by considering that the assemblage with the Plexiglas device affected the drug release rate at the pH value where the drug solubility was lower by slowing down the matrix dissolution process. A similar behavior was reported by Bettini et al. [22] in erosion-controlled matrices containing a poorly soluble drug.

## 4. Conclusions

The two studied complexes released the drug with different mechanism indicating two different drug/polymer interaction strengths.

The comparison between the drug release behavior of the complexes and the relevant physical mixtures indicates that diltiazem gives rise to a less soluble and more stable complex with carrageenan than metoprolol.

The less stable metoprolol complex affords an erodible matrix, whereas the stronger interaction between diltiazem and carrageenan results in a poorly soluble, slowly dissolving matrix.

As far as the morphological matrix modifications are concerned, the position of three fronts was visualized in matrices prepared with noncolored components.

The diltiazem-carrageenan matrix behaves as an extremely slow eroding matrix: the front of water moved inward, while the diffusion front position remains fairly constant. Metoprolol matrices erode almost completely in 8 hours giving rise to the synchronization of water, diffusion, and matrix fronts in all the dissolution media studied.

It can be concluded that the different stability of the studied complexes affords two distinct drug delivery systems: in the case of MTP, the dissociation of the complex, as a consequence of the interaction with dissolution medium, affords a classical soluble matrix type delivery system; in the case of DTZ, the dissolving/diffusing species is the complex itself because of the very strong interaction between the drug and the polymer. In this case, the tablet has to be considered as a monolith constituted by a unique insoluble compound rather than a matrix system.

## Figures and Tables

**Table 1:** Values of the exponent *n* and relevant correlation coefficients calculated with (1) for drug release profiles from different MTP containing matrices in different media. Standard deviation in parenthesis (*n* = 3).

Matrix/medium	*n*	*R* ^2^
MTP-CRG/water	0.77 (0.008)	0.999
MTP-CRG/pH 1.2	0.64 (0.001)	1
MTP-CRG/pH 6.8	0.68 (0.0001)	0.999
MTP-HPMC FM/water	0.62 (0.008)	0.996
MTP-CRG FM/water	0.89 (0.001)	0.999
